# Biodiversity Analysis of Forest Litter Ant Assemblages in the Wayanad Region of Western Ghats Using Taxonomic and Conventional Diversity Measures

**DOI:** 10.1673/031.007.0601

**Published:** 2007-01-25

**Authors:** Anto Anu, Thomas K. Sabu

**Affiliations:** Litter Entomology Research Unit, P.G. and Research Department of Zoology, St. Joseph's College, Devagiri, Calicut, Kerala, India-673 008

**Keywords:** evergreen, deciduous, Shola evergreen forests, litter ant biodiversity, taxonomic diversity indices

## Abstract

The diversity of litter ant assemblages in evergreen, deciduous and Shola evergreen (Shola) forest vegetation types of the Wayanad region of the Western Ghats was assessed employing conventional and taxonomic diversity indices. Non-dependence on quantitative data and the ability to relate the phylogenetic structure of assemblages with ecological conditions of the habitat, and to ascertain priorities for conservation of habitats, makes non-parametric taxonomic diversity measures, such as variation in taxonomic distinctness Λ^+^ and average taxonomic distinctness Δ^+^, highly useful tools for assessment of litter ant biodiversity.

Although Δ^+^ values saturated leading to closer values for the 3 litter ant assemblages, Λ^+^ proved to be a more dependable index. Evenness in taxonomic spread was high in ant assemblages in deciduous forests and low in evergreen forests compared to the regional master list. Low Λ^+^ of ant assemblage in deciduous forests indicates that among the 3 forest vegetation types, deciduous forests provided the most favorable habitat conditions for litter ants. Low evenness, as is indicated by Λ^+^ in evergreen forests, was attributed to the presence of a group of taxonomically closely related ant assemblage more adapted to prevail in moist and wet ecological conditions.

## Introduction

With a wide array of bioclimatic and topographic conditions, the Western Ghats has a high level of biodiversity and endemism and at the same time it is one of the most threatened regions, that has earned it the status of a biodiversity ‘hotspot’ (Bossuyt et al. 2004; Myers et al. 2000). Though the floral and vertebrate faunal diversity found in the region are well documented, little is known of the litter ant distributional patterns or assemblages in the different vegetation types of Western Ghats forests. Forests in the transitional Wayanad region of Western Ghats consist of 3 primary vegetation types; moist deciduous forests, evergreen forests and Shola evergreen (Shola) forests. Low elevation evergreen forests dominated by dipterocarps constitute the most threatened habitat in the Wayanad region and its continuum along the Western Ghats has been fragmented (Nair 1991; Pascal 1991). It is well known that structural changes in vegetation and related variations in site temperature, rainfall and food resource availability control litter ant species richness and diversity (Alonso 2000; Brühl et al. 1999; Olson 1994).

Distinct dissimilarity between the litter ant assemblages of Shola evergreen and deciduous forests in the Wayanad region in relation to the forest vegetation specific abiotic factors has been recorded. Litter ant species specific to the evergreen forest represent species adapted to a cool, moist climate, and the species specific to deciduous forests represent species adapted to open dry, hot climates (unpublished observations). Together with their relative stability, moderate diversity and sensitivity to microclimate, litter dwelling ant communities fulfil all criteria of a potential taxon to be used as indicators of ecosystem modifications in various habitats (Kremen 1992; Kremen et al. 1993; Lawton et al. 1998; Agosti et al. 2000).

In an analysis of community diversity of colonial organisms such as ants, what is taken into account is the frequency of occurrence of species in each sample, and not the number of individuals of each species encountered during the inventory, as a single sample may contain an extreme abundance of a rare species. Usage of occurrence data instead of individual-based analysis leads to the situation where only one species occurrence is counted in a sample even when two colonies may be present and some information on abundance is lost (Leponce et al. 2004; Brühl et al. 2003; Longino 2000; Pfeiffer et al. 2003). Even with a combination of pitfall traps and Winkler extractors, which is considered as the best method for collecting soil and litter dwelling ants, only <50% of the total species present could be captured (Delabie et al. 2000). Hence, even with all precautions one can not be sure of the efficiency of the sampling effort in litter ant faunal inventories.

Another difficulty in sampling litter ants is that litter fauna are locally very numerous, with a wide range of mobility requiring enormous sampling efforts where complete enumeration is not possible as fauna may shift in relation to microclimate/environmental factors (Bestelmeyer 2000; Bruhl et al. 1998; Elmes and Wardlaw 1982). This may lead to variations in evenness and abundance of ant species in samples with regard to environmental factors. Since conventional indices of community diversity are very sensitive to disparities in sampling effort, they are of little value in comparative biodiversity measures unless the sampling Methods, sample sizes and habitat types are carefully controlled (Clarke and Warwick 2001). Except at high levels of disturbances, species diversity based on evenness and richness do not provide a reliable measure of important changes in biodiversity (Warwick and Clarke 2001). In most cases when conventional indices are used one is not sure whether or not the variations in diversity arise from sampling inadequacy (Warwick and Clarke 1998, 2001; Rogers et al. 1999).

Little attention has been devoted to analyse the ways in which habitat modifications affect phylogenetic structure at local or regional scales, and the extent to which properties of phylogenetic structure can be used as measures of biodiversity (Clarke and Warwick 2001). The phylogenetic structure of any assemblage is clearly important, and an assemblage comprising a group of closely related species must be regarded as less ‘biodiverse’ than an assemblage of the same number of more distantly related species, for example, when they all belong to different phyla (Clarke and Warwick 2001). Hence, interpretations of biodiversity using conventional evenness/richness-based species diversity measures alone would be of little use. If we continue with conventional measures of diversity for monitoring the spatial and temporal variations in biodiversity, changes in biodiversity may go undetected until very advanced stages of biodiversity loss and environmental degradation is reached (Clarke and Warwick 1998).

**Figure 1.**
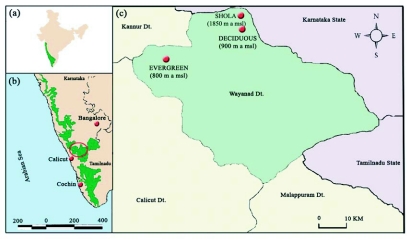
(a) Map of India showing the location of the Western Ghats, (b) Western Ghats and (c) study site in the Wynad region of Western Ghats.

Among the number of biodiversity measures developed recently, four indices based on taxonomic relatedness between the species or individuals (Warwick and Clarke 1995, 1998 and 2001; Warwick et al. 2002) are rated as most promising for biodiversity assessment (Feral et al. 2003; Magurran 2003). Taxonomic relatedness-based diversity indices are relatively insensitive to disparities in sampling effort, and additionally provide more intuitive information than conventional ‘species’ indices (Warwick and Clarke 2001; Warwick et al. 2002; Price 2002). In the present study, we analyzed the litter ant assemblage properties of evergreen, deciduous and Shola evergreen forests in the Wayanad region of Western Ghats, employing conventional and taxonomic relatedness based indices and recorded: 1) how ant assemblage properties vary in relation to forest vegetation type; 2) how interpretations based on taxonomic diversity differ from interpretations based on standard diversity indices; and 3) the practical utility of taxonomic diversity based measures in determining the conservation value of forest vegetations and the ant assemblages they support.

## Methods

### Study Site

The study was conducted in the evergreen, deciduous and Shola forest vegetation types in the Wayanad region of Nilgiri biosphere in Kerala state, in Western Ghats (Figure 1). The Western Ghats is a string of low-lying mountains along the west coast of the Indian subcontinent rising from a relatively narrow strip of coast at the western border, reaching up to a height of 2800 m before they merge to the east with the Deccan plateau at an altitude of 500–600 m (Bhatt and Magurran 2006). Biogeographically, the Wayanad region of Western Ghats is a transitional zone between the moist forests of the south Western Ghats and the dry forests of the northern region, harboring habitat-restricted endemic species, as well as disjunct populations of species that are found in both regions (Pascal 1988; WWF 2001). Approximately 1000 sq km of the original vegetation remain in the Wayanad region consisting of deciduous, evergreen and Shola forests excluding the Camel's hump mountains (Nair 1991). Deciduous and Shola forest study sites are on the south-eastern part, and the evergreens are on the western part of the Wayanad plateau. Montane temperate evergreen forests, locally known as Shola forests, are located in the upper region, 1350 m above sea level, deciduous forests are located in the mid elevations 950 m above sea level at Brahmagiri, and wet evergreen forests are located in the lower elevations, 800 m above sea level at Chanthanathode in the north Wayanad Western Ghats ecoregion 11° 50′ N latitude and 75° 49′ E longitude. Terrain is even at deciduous and evergreen forests and undulating in the Shola region. The northeast monsoon from October to November supplements the June to September southwest monsoon in the entire region. There is considerable variation in the total annual rainfall received in these regions. The deciduous forest region receives rainfall of around 2000 mm/yr and evergreen region receives 3500–6000 mm of rainfall. Rainfall figures from the Shola region are not available.

### Sampling protocol

A preliminary transect, following the standard ‘ants of leaf litter’ protocol (Agosti et al. 2000) was conducted in the evergreen forest at Chanthanathode in January 2004. A 200 m long line transect proposed in the this protocol was traced at intervals of 10 m. The leaf litter present inside a 1 m2 quadrat was collected, sifted and put in a bag. The sifted material was brought back to the field laboratory in collecting bags and fauna was extracted with a mini-Winkler apparatus (Fisher 1998; Bestelmeyer et al. 2000) for 24 h. Ants were hand picked, and transferred to labeled containers of 70% alcohol. For deciduous and Shola forests, data from the ant faunal inventories conducted during January 2004 (unpublished observations) were used.

### Species identification

Collected ant species samples were identified primarily based on Bolton (1994) and Fauna of British India, Bingham (1903). Voucher specimens were temporarily deposited with the Museum of the Department of Zoology, St. Joseph's College, Calicut, Kerala.

### Data analysis

The frequency of incidence of species was used as measure of abundance of leaf-litter ants (Leponce et al. 2004; Brühl et al. 2003; Longino 2000; Pfeiffer et al. 2003). The number of individuals of each species present was not recorded. A species present in all 20 samples was given an incidence value of 20. Hence the abundance data was non-quantitative incidence data rather than quantitative abundance data obtained from the numbers of individuals present.

Ant biodiversity in the 3 forest vegetation types were analysed with both ‘traditional’ indices and taxonomic relatedness-based biodiversity indices. The ‘traditional’ alpha diversity indices were: number of species (S), Simpson's diversity index (1/D), Simpson's evenness index (1- 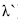) (Simpson 1949) and Shannon's diversity index (H', using loge) were used (Shannon and Weaver 1949). Although there are many possible indices that can be used to depict diversity, overdependence of these indices on sampling effort is stated as one of the fundamental difficulties in all fields of biodiversity assessment (Warwick and Clarke 2001). Simpson's index is a notable exception making it one of the most meaningful and robust traditional diversity measures available (Magurran 2003; Lande 1996). The Shannon-Weaver index was also determined as it is has been widely used by ecologists.

Beta diversity was analysed using the Bray-Curtis similarity index using presence/absence data. Cluster analysis was done following hierarchical agglomerative clustering (Bray and Curtis 1957).

Taxonomic diversity was analyzed with 4 taxonomic relatedness based indices: average taxonomic diversity Δ, average taxonomic distinctness based on abundance data Δ^*^, average taxonomic distinctness based on incidence data Δ^+^, and variation in taxonomic distinctness Λ^+^ (Clarke and Warwick 2001, Warwick *et al*. 2002). Δ and Δ^*^ are parametric measures based on abundance data and Δ^+^ and Λ^+^ are non-parametric measures based on presence/absence data.

A regional master list of litter ants in Wayanad was compiled using the data from Sabu (2005), unpublished observations and the present study in evergreen forests. A randomization test was done to detect a difference in the average taxonomic distinctness and variation in taxonomic distinctness, for any observed set of species, from the ‘expected’ Δ^+^ and Λ^+^ value derived from the regional master species list (Clarke and Warwick 1998). The null expectation was that the species present at any one place or time behave like a random selection from the regional species selection pool.

Five taxonomic levels namely, species, genus, tribe, subfamily and family were considered. Branch lengths between the taxonomic classes were defined following the standardization proposed by Warwick and Clarke (2001). We assumed equal step lengths between each successive taxonomic level, setting the path length ω to 100 for 2 species connected at the highest (taxonomically coarsest) possible level. So the weights are ω = 20 (species in the same genus), ω = 40 (same tribe but different genera), ω = 60 (same subfamily but different tribe) and ω =80 (same family but different subfamily).

**Figure 2.**
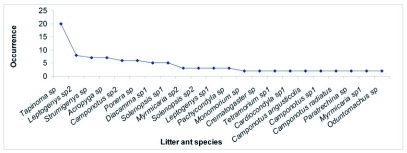
Rank based abundance (incidence) plot of the litter ant community in the evergreen forest site.

Rank abundance of the evergreen ant assemblage was plotted with incidence of ants against ranks (Whittaker 1965). Simpson's diversity index was calculated with Estimates 7.5 program (Colwell 2005). All other diversity analysis was done with PRIMER 5 software version 5.2.9 (Clarke and Gorley 2002). Variances of traditional diversity indices and quantitative taxonomic diversity indices Δ^+^ and Λ^+^ were computed using PAST ver 1.43 (Hammer et al. 2001). Variances of qualitative taxonomic diversity indices values Δ^+^ and Λ^+^ with respect to the master list values were estimated by drawing 95% confidence funnels using PRIMER package (Clarke and Gorley 2002). Analysis is summarized by 2-dimensional Δ^+^, Λ^+^ plots, placing the real Δ^+^ and Λ^+^ pairs in context of 95% probability envelopes from simulated samples of comparable sizes drawn from the master list of litter ant assemblage from Wayanad forests. Data points outside the relevant 95% contour or ellipse plots imply statistical evidence of ‘departure from expectation’ for those studies (Clarke and Warwick 2001).

**Table 1.**
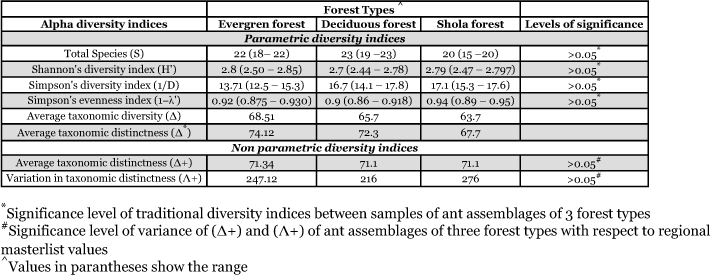
Conventional and taxonomic diversity indices of the forest litter ant assemblages and their variances in three major forest vegetation types in the Wynad region of Western Ghats.

### Results

**Figure 3.**
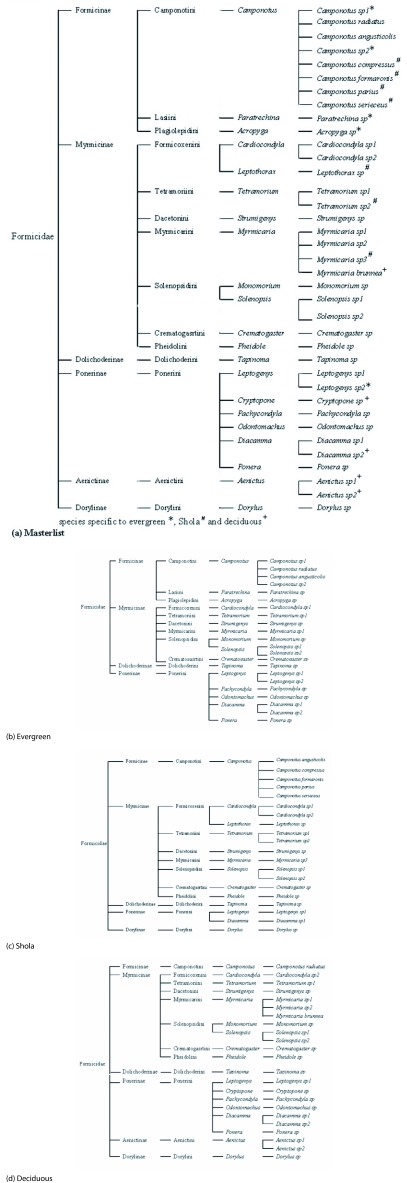
Taxonomic (phylogenetic) relationships of the litter ant community in the Wayanad forests: (a) masterlist, (b) evergreen, (c) Shola, (d) deciduous forests.

22 ant species from 16 genera, 11 tribes and 4 subfamilies were found in the evergreen forest study site (Figure 2); *Tapinoma sp* was the dominant species. The Formicinae was the most highly speciose subfamily in evergreen forests (Figure 3b); Two Formicinae tribes (Laginii and Plagiolepidini) were reported only from evergreen forests; Dorylinae and Aenictinae subfamilies were not found in evergreen forests. Aenictinae were present only in deciduous forests (Figure 3d). Ponerinae were less speciose in Shola forests in comparison to their high speciosity in evergreen and deciduous forests (Figure 3). *Camponotus* was the most speciose genus in evergreen and Shola forests, while the genus *Myrmicaria* was most speciose in deciduous forests (Figure 3).

**Figure 4.**
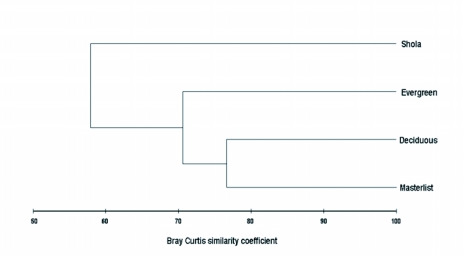
Dendrogram based on hierarchical agglomerative clustering (group-average linking) of litter ant faunal assemblages in 3 forest vegetation types of Wynad.

Conventional diversity indices provided contrasting Results whereas the parametric taxonomic diversity indices provided a uniform pattern (Table 1). Shannon's diversity index (H') recorded high and equal diversity in both evergreen and Shola forests, but Simpson's diversity index (1/D) recorded higher diversity in Shola forests. Simpson's evenness showed high and equal evenness at all sites. Both parametric taxonomic diversity indices Δ and Δ^*^) recorded high diversity in evergreen litter habitats and lowest diversity in Shola forest. Non-parametric taxonomic diversity indices Δ^+^ and Λ^+^ showed identical Δ^+^ values and dissimilar Λ^+^ values for the 3 ant assemblages. Variation in taxonomic distinctness Λ^+^ was highest in Shola forests and lowest in deciduous forests (Table.1). The Bray Curtis similarity coefficient showed high similarity between the regional master list and deciduous ant assemblages, and between deciduous and evergreen assemblages. The distinct dissimilarity of the ant assemblages in Shola forests from other forests and the regional litter ant pool is obvious in the dendrogram (Figure 4). The deciduous forest has the highest, and Shola has the lowest, number of representative species in common with the Wayanad regional species list of litter ants.

**Figure 5.**
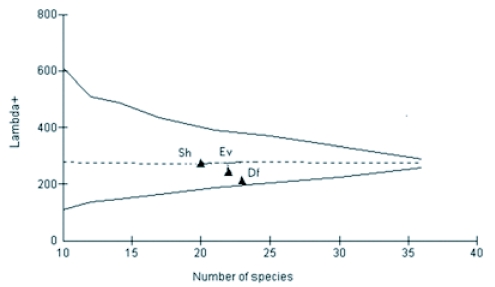
Measured values of Λ^+^ of 3 litter ant assemblages in Wayanad forests, plotted against the number of species as points on the simulated 95% confidence funnel (dashed lines, based on random selections from the total Wayanad species list) and theoretical 95% confidence funnel (continuous line based on variance formula). Sh — Shola, Ev —evergreen, Df-deciduous forests.

Comparative analysis of the ant species list and taxonomic structure of the assemblages provided the following details (Figure 3). 37 species from 21 genera, and 14 tribes belonging to six subfamilies, were recorded from Wayanad forests. 23 ant species belonging to 18 genera, 12 tribes and 6 subfamilies were present in deciduous forest, and 20 species from 13 genera, 11 tribes and 5 subfamilies in the Shola forest. 8 species were common to all three sites; 5 were specific to evergreen forest, 5 to deciduous forest and 7 to Shola forest.

**Figure 6.**
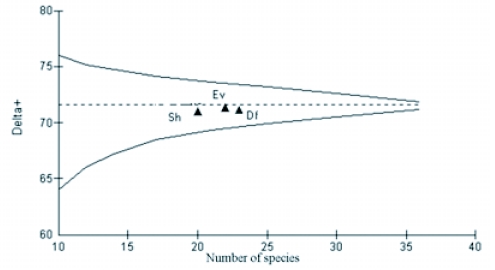
Measured values of Δ^+^ of 3 litter ant assemblages in Wayanad forests, plotted against the number of species as points on the simulated 95% confidence funnel (dashed lines, based on random selections from the total Wynad species list) and theoretical 95% confidence funnel (continuous line based on variance formula). Sh — Shola, Ev -evergreen, Df- deciduous forests.

**Figure 7.**
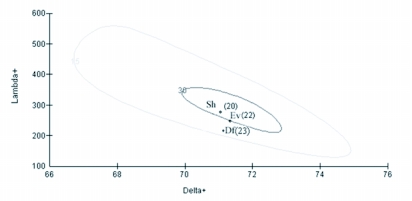
Bivariate plots showing 95% probability envelopes of variation in taxonomic distinctness and average taxonomic distinctness based on a regional master ant species list. Observed number of species in parenthesis. Sublists of size 20 and 30 are simulated, spanning the observed range of list sizes for ant assemblages. Sh - Shola, Ev - evergreen, Df - deciduous forests.

Values of both average taxonomic distinctness Δ^+^ and variation in the taxonomic distinctness Λ^+^ for ant species from all forest vegetation types fell within the 95% limits of probability of funnel for the appropriate numbers of species randomly drawn from the regional species pool (each based on 1000 random selection) (Figures 5 and 6). Fitted 95% probability contours of the joint Δ^+^ and Λ^+^ distributions and the resultant ellipse plot showed that all the real points fell within the contour showing no significant variation in the taxonomic structure of the 3 ant assemblages from the regional master list (Figure 7).

## Discussion

Taxonomic evenness properties of litter ant assemblages in the 3 forest vegetations within same geographical region varied considerably. This variation was linked to litter physical conditions characteristic of the respective forest vegetation type. Traditional diversity indices, which do not consider the relatedness between species and with their overdependence on numerical properties of assemblages, lack the capacity to link diversity values with habitat conditions. The present study shows that the non-parametric taxonomic diversity measure, Λ^+^, provides a more meaningful assessment of the diversity of ant assemblages by using the taxonomic relatedness properties of ant assemblages with a phylogenetic approach. Thus, Λ^+^ is able to relate the resulting pattern with habitat ecological conditions that is not possible with conventional diversity indices. In addition, Λ^+^ has proved to be a more dependable diversity index than Δ^+^, as the latter does not consider the variation in the evenness in taxonomic distribution. Ant assemblages in deciduous forests with warm and dry litter habitat conditions that are more conducive for foraging activities of litter ants resulted in high evenness (low Λ^+^), while assemblages in the cold, wet/moist litter floor in Shola forests with less favorable conditions (Brühl et al. 1998; Samson et al. 1997; Olson 1994) recorded low evenness in taxonomic distribution (high Λ^+^). High diversity in evergreen forests indicated by both parametric taxonomic diversity measures, Δ and Δ^*^, are more appropriate than the contrasting trends shown by the richness and evenness based conventional indices in evergreen and Shola forests, as the conventional quantitative taxonomic diversity indices consider taxonomic relatedness properties along with richness and evenness.

The abundance of *Tapinoma sp* in the litter floor of all forest vegetation types, in both wet and dry habitats, suggests that they are the best adapted ant taxon in the Wayanad region. This observation is in accordance with earlier report of this taxon as the common group in the region irrespective of the vegetation types in Wayanad forests (Sabu 2005). The presence of two wet rainforests preferring genera, *Acropyga sp* and *Paratrechina sp* (Shattock and Barnett 2001), which were exclusively found in the evergreen forest site is indicative of the influence of litter habitat conditions in determining the habitat preference of ants. Low numbers of true litter ants (Ponerinae) in Shola forests is attributed to their low preference for cold moist habitats (Brühl et al. 1998). Conventional, and the parametric taxonomic relatedness based diversity indices Δ, Δ^*^), presented contrasting information about the diversity of litter ant assemblages in 3 forest types.This difference arises because there is no single universally applicable index incorporating all the essential elements of an ideal biodiversity index (Margules and Pressey 2000). An ideal index, should be the the sum of taxonomic, numerical, ecological, genetic, historical and phylogenetic diversity and the stronger the intercorrelations among these different diversity measures the more robust such an approach would be (Van der spoel 1994; Purvis and Hecter 2000). Lack of such an ideal index often leads to contrasting interpretations and prioritisation of assemblages and habitats, as assemblages and habitats identified as highly diverse by one index may become less diverse when measured by another index (Price 2002). Since taxonomic relatedness based parametric measures Δ, Δ^*^ include more of the essential requirements of an ideal biodiversity index, we consider the high diversity indicated by Δ and Δ^*^ in evergreen forests more appropriate than the contrasting trends shown by conventional diversity indices.

Similar Δ^+^ values for the 3 assemblages indicates that all have similar assemblage properties and are in a late stage of succession, consisting of species belonging to a wide range of phyla. But a closer look at their phylogenetic structure shows that Δ^+^ did not consider the variation in the evenness of taxonomic species distribution that resulted in closer Δ^+^ values for all the 3 assemblages. Closer values arises because, though Δ^+^ is effective in contrasting situations with a restricted number of higher taxa, the presence of many speciose genera tend to saturate Δ^+^ leading to closer values (Izsak and Price 2001). By truncating the phylogenetic tree and analyzing the variations in Δ^+^ we observed that the presence of 6 subfamilies, and one subfamily (Aenictinae) specific to deciduous forests, raised Δ^+^ in deciduous forests. But the presence of two speciose subfamilies (Myrmicinae and Ponerinae) negated the advantages resulting in low Δ^+^. Similarly, the presence of two tribes reported only from evergreen forests (Lasiini and Plagiolepidini) raised Δ^+^ in evergreen forests, but the presence of a highly speciose genus *Camponotus* and absence of 2 subfamilies (Aenictinae and Dorylinae) negated these advantages. Hence the 3 assemblages have closer Δ^+^ values. Clarke and Warwick (2001) mentioned the inability of Δ^+^ to recognise the variations in taxonomic evenness as its major drawback in certain estimations and recommended the use of Λ^+^, which considers the variation in the evenness of taxonomic distribution of the assemblage.

Closer Λ^+^ values in evergreen and Shola evergreen forests, in comparison to the regional ant pool, indicate similarity in the taxonomic structure of litter ant assemblages in ecologically more similar wet evergreen and Shola evergreen forests. It is an instance of similar Linnaean taxonomic tree structure in ecologically similar habitats (Warwick et al. 2002). Contrastingly, the conventional beta similarity index (Bray Curtis) detected higher similarities between the ant assemblages of ecologically dissimilar deciduous and evergreen forests.

Λ^+^ is particularly relevant in conditions of larger spatial scales such as forest biomes where habitat heterogeneity is likely to influence faunal diversity patterns. High Λ^+^ and lower taxonomic spread is a feature of degraded environments (Warwick et al. 2002, Clarke and Warwick 2001). For all groups of organisms specific taxa attain their highest diversity in particular habitats, but if certain habitat types are absent from an area then some groups of species become under represented and others become over represented compared with the regional picture (Warwick and Clarke 2001). Normally, the species in disturbed environments would be of a closely related assemblage with similar habitat requirements and this would normally be manifested as a decrease in Δ^+^ and rise in Λ^+^ values. But in some cases, as observed with Shola and evergreen ant assemblages, the Δ^+^ failed to differentiate the taxonomic relationship of closely related assemblages and remained stable but Λ^+^ rose (Clarke and Warwick 2001). The lower Λ^+^ for deciduous forests shows that among the 3 forest vegetations, the ant assemblages in deciduous forests had the highest evenness in distribution of taxa across the taxonomic tree indicating a stable distribution and a more diverse assemblage in the region. Although high Λ^+^ and hence lower taxonomic spread in Shola and evergreen forests indicates the presence of a phylogenetically closely related ant assemblages with similar ecological adaptations to survive in a wet/moist litter that is less conducive for litter ants, it should not be considered as an indication of habitat degradation as both the evergreen and Shola forests are well protected, undisturbed mature forests (Nair 1991).

Litter ant assemblages of Shola and evergreen forests consist predominantly of functional guilds adapted to exist in wet/moist litter habitats (unpublished observations). Similar ecological conditions namely, wet conditions at Shola and evergreen forests (Sabu 2005, and unpublished observations) result in the presence of ants from closely related taxonomic groups in which all genera would be relatively more species rich (for example the genus *Camponotus* with 5 species in Shola, 4 species in evergreen, and only 1 in deciduous, compared to 6 in the Wayanad region) leading to high unevenness in the distribution of taxa. Warwick and Clarke (2001) reported the same situation in areas where certain habitats were absent (i.e. low habitat diversity) so that taxa normally found in these missing habitats were absent, leading to higher Λ^+^ values than in situations with the full range of habitat types. Taxonomically closely related ant assemblages in the Shola and evergreen forest floors, even with low evenness values, demand the highest conservation attention due to their adaptedness to survive in the least favorable wet/moist litter floor of the few remaining evergreen forests in the Wayand region (Nair 1991; WWF 2001).The presence of rare *Acropyga* species reported exclusively from the moist evergreen forests is a testimony to this observation. The high evenness of the ant assemblages in the deciduous forests is linked to the establishment of a taxonomically well-spread ant assemblage facilitated by the presense of open and dry litter habitats conducive for litter ants (Brühl 1999; Olson 1994; unpublished observations).

The Δ^+^ and Λ^+^ values of none of the ant assemblages were outside the expectation as their values did not fall below the 95% limit of the simulated distribution indicating that none of the assemblages are with low taxonomic distinctness in comparison to the regional pool. Although the Bray Curtis similarity coefficient could measure the similarity of the ant assemblages between habitats based on the shared and non-shared species, it was unable to relate the resulting pattern with habitat ecological conditions and provide meaningful interpretations. Multivariate measures, such as MDS, are considered the most sensitive measure in terms of detecting changes in community structure compared to univariate biodiversity measures, but they do not indicate deleterious changes that are usually achieved by linking community structure to univariate environmental measures (Clarke and Warwick 2001).

This study supports the view of Magurran (2003) and Warwick and Clarke (2001) that Δ^+^ and Λ^+^, with their appealing sampling properties, non-dependence on quantitative data, and their consideration of the relatedness of the species present, are of great practical utility in diversity analysis. The non-dependence on quantitative data and intuitive capacities of these indices to relate to litter habitat conditions is of great significance in litter ant diversity analysis. It is necessary to rethink the continued usage of conventional evenness and richness based alone in biodiversity assessments in view of the observations of Harper and Hawksworth (1994) on the role of biodiversity indices. A measure of biodiversity of a habitat should say something about how different the inhabitants are from each other (Harper and Hawksworth 1994). To simply say whether the inhabitants belong to same genus, tribes, species does not serve any purpose in biodiversity assessments. The present study shows the ability of taxonomic relatedness-based diversity indices to relate the phylogenetic structure of ant assemblages with habitat ecological conditions and habitat heterogeneity. Their ability to evaluate priorities for conservation of habitats makes them more attractive than the conventional diversity indices for future biodiversity assessments. However, we stress that any conclusions about the utility of taxonomic indices drawn from forest litter ant assemblages alone must be tentative, pending compilation and analysis of comprehensive datasets and the taxonomic diversity properties of other fauna and flora.

### Implications of our findings

Taxonomic diversity of the ant assemblages varied between the forest vegetations and the role of the vegetation type in deciding the phylogenetic structure of ant community would not have been noticed if conventional indices had been used. Non-dependence of sampling, the utility of non-qunatitative historical data for comparisons, and the ability to relate with habitat quality makes taxonomic distinctness measures more convenient tools for diversity assessments. Non-dependence of sampling is of great significance in diversity analysis of social insects considering the practical difficulties enumerated in detail by Leponce (2004). The current study highlights the practical utility of taxonomic relatedness-based biodiversity indices in diversity assessment and their superior qualities in comparison to conventional indices.
